# Digital health literacy of adolescents: A scoping review protocol

**DOI:** 10.1371/journal.pone.0348021

**Published:** 2026-05-06

**Authors:** Hannah Linney, Ken van Someren, Sinead Keogh

**Affiliations:** 1 Department of Enterprise and Technology, Atlantic Technological University, Galway, Ireland; 2 School of Health, Sport Science and Nutrition, Atlantic Technological University, Donegal, Ireland; University of Saskatchewan, CANADA

## Abstract

**Introduction:**

Adolescence is a key developmental period in which new health behaviours are often initiated that track into adulthood. In the age of digitisation, adolescents are frequently exposed to and actively seek health information from digital sources. Widespread misinformation necessitates the ability to critically evaluate the reliability of sources, a key component of digital health literacy. Digital health literacy, sometimes called e-health literacy, is considered a determinant of health. Evidence suggests an association between digital health literacy and health behaviours among adults; however, the concept has been sparsely investigated among adolescents. This protocol outlines a proposed scoping review that aims to identify, map and synthesise the currently available literature relating to the digital health literacy of adolescents.

**Objective:**

The objective of the proposed scoping review is to understand the extent, breadth and type of evidence available regarding the digital health literacy or e-health literacy of adolescents.

**Methods:**

A scoping review of literature will be conducted in accordance with the methodological framework proposed by Arksey and O’Malley, with refinements suggested by Levac et al and guided by the Joanna Briggs Institute (JBI) Manual for Evidence Synthesis. Reporting will follow the Preferred Reporting Items for Systematic Reviews and Meta-analysis extension for Scoping Reviews (PRISMA-ScR). Searches for relevant studies have been conducted across five electronic databases (PubMed, CINAHL, Web of Science, Education Source and Scopus). Title and abstract screening, which has commenced, will be conducted primarily by the lead reviewer, with independent verification of a subset of records to enhance rigour and minimise selection bias. This will be followed by full text screening using Covidence software. The PCC (Population, Concept and Context) framework guided the development of the inclusion and exclusion criteria for study selection. A manual search of references of included studies will be conducted to identify further studies for inclusion. Data will be extracted and analysed using a descriptive mapping approach.

**Inclusion criteria:**

Published quantitative and qualitative studies exploring the digital health literacy, or e-health literacy of adolescents aged 10 to 19 years will be included. Eligible study designs include randomised controlled trials, non-randomised controlled trials, pre-post studies, prospective and retrospective cohort studies, case-control studies and analytical cross-sectional studies. This review will also consider descriptive observational study designs including case series, individual case reports and descriptive cross-sectional studies for inclusion.

**Discussion:**

The proposed scoping review aims to map how digital health literacy is defined and measured among adolescents, providing greater conceptual clarity within the emerging field. By identifying patterns, inconsistencies, and gaps in the literature, the findings are expected to inform future research priorities, scale development, and the design of targeted interventions in educational and public health settings. Consistent with the scoping review methodology, a risk of bias assessment will not be conducted however the systematic approach will map the available evidence and form the basis for future research. This scoping review protocol has been preregistered on the Open Science Framework (OSF Registries, available at: https://osf.io/xsvpz/).

## Introduction

Health literacy is defined as ‘people’s knowledge, motivation and competencies to access, understand, appraise and apply health information to make judgements and take decisions in everyday life concerning healthcare, disease prevention and health promotion to maintain or improve quality of life during the life course’ [1]. According to Nutbeam’s hierarchical model, health literacy consists of three components: functional health literacy (basic literacy skills and health knowledge), interactive health literacy (ability to communicate one’s health needs and understand information) and critical health literacy (skills to critically evaluate health information and apply it to different contexts) [2]. Health literacy is considered a key determinant of health, a potential mediator for demographic factors and is associated with positive health behaviours in adolescents [3,4]. Low health literacy is associated with poor health outcomes, reduced interaction with preventative services and reduced quality of life [4]. Poor health literacy is highly prevalent in adults across Europe and the USA, however the health literacy of adolescents is less well understood [5,6].

With the widespread emergence of new technologies, a related concept of digital health literacy, sometimes referred to as e-health literacy, has emerged. It is defined as the ability to seek, find, understand and appraise health information from a digital source to apply it to maintain good health and overcome health challenges [7]. Digital technologies have infiltrated every aspect of modern life. In the U.S, 95% of adolescents report using the internet daily, similarly 88% of respondents in 2021/2022 HBSC survey report using social media at least daily [8,9]. Adolescents are exposed to a large amount of diverse health information of varying quality and trustworthiness from a range of digital sources such as social media and internet websites [10]. Evidence suggests they also frequently actively seek health information on topics such as nutrition, mental health and sexual health from digital sources [11,12]. The abundance of easily accessible information available can be a source of empowerment for adolescents to become active agents in their own health [11–13]. The largely unregulated nature of the social media and internet sources, however, necessitates the ability to appraise the quality and reliability of information accessed, as demonstrated by the Covid 19 infodemic [14]. Adolescents may not have the necessary skills to critically evaluate reliability of information, potentially impacting their decision making and overall health [15]. Thus, further understanding is needed on the digital health literacy of adolescents.

Adolescence is a key developmental period involving rapid physical, emotional, cognitive, and social development [16]. It is characterised as a period of increased autonomy and independence [17]. During adolescence the limbic subcortical systems, the area of the brain responsible for emotions and reward processing, matures earlier than the prefrontal cortex, which is responsible for decision-making and impulse control [18]. Adolescents are therefore susceptible to engaging in risky behaviours. Initiation of problematic behaviours such as smoking, alcohol consumption and substance use is highest in this period and evidence suggests these behaviours track into adulthood, contributing to the epidemic of non-communicable disease [17,19]. It is estimated that approximately 70% of premature deaths in adulthood can be attributed to behaviours established in adolescence, such as obesogenic diets, smoking, alcohol consumption and physical inactivity [16]. According to the Health Behaviour in School-Aged Children (HBSC) 2021/2022 survey, less than 25% of boys and less than 15% of girls reported achieving recommended levels of physical activity levels [20]. Less than 40% report eating fruit and vegetables daily and more than 20% were classified as overweight or obese, an increase in more than a third of locations since the 2018 survey [20]. Prevalence of e-cigarette use increased in all but two regions in the most recent survey and prevalence of reported rates of unprotected sex has increased by 9% among boys and 6% among girls in 2022 compared to 2014 [21,22]. Targeted health promotion initiatives for the demographic should therefore be a public health priority. Digital health literacy is considered a super determinant of health, associated with health behaviours in adults, although it is poorly understood among adolescents [23].

The objective of the proposed scoping review is to assess the extent, breadth and type of literature exploring the digital health literacy of adolescents, investigating how it is defined and measured. This is the first systematic effort to examine the digital health literacy specifically of this demographic. The scoping review methodology was chosen as this is an emerging field of research that is poorly understood. A preliminary search of PubMed, the Cochrane Database of Systematic Reviews, Prospero and JBI Evidence Synthesis was conducted and no current or underway systematic reviews or scoping reviews on the topic were identified.

This protocol comprehensively outlines the chosen methodology for conducting the proposed scoping review. The aim of publishing this protocol is to ensure transparency and limit bias in the reporting of the proposed scoping review. Deviations made from this protocol will be reported and justified in the scoping review.

### Review question

What is the extent and breadth of available literature on the digital health literacy of adolescents?

How is digital health literacy defined and measured in the included studies?

### Inclusion criteria

#### Participants.

This scoping review will consider studies that measure the digital health literacy, sometimes referred to as e-health literacy, of adolescents. Studies will be included if participants are aged between 10 and 19 years, in accordance with the WHO definition of adolescents [24]. Studies that include a broader age range will be considered for inclusion if the adolescent population are presented as a subset.

#### Concept.

The concept of interest is digital health literacy or e-health literacy. E-health literacy was first introduced by Norman and Skinner as the ability to look for, find, understand, appraise and apply health information from electronic sources to address or solve a health problem [25]. Some sources use the terms e-health literacy and digital health literacy interchangeably, while other studies consider digital health literacy as an expansion of e-health literacy encompassing broader digital environments [23]. For the purposes of the proposed review studies that measure digital health literacy or e-health literacy of adolescents will be included. Interventions aimed at improving digital health literacy and e-health literacy will be included where measurement of digital health literacy or e-health literacy is reported as an outcome.

#### Context.

Studies that explore digital health literacy in any context, including but not limited to schools, healthcare and the community will be eligible for inclusion.

#### Types of sources.

The proposed scoping review will consider published quantitative and qualitative research studies. Eligible study designs include experimental and quasi-experimental studies (e.g., randomised controlled trials, non-randomised controlled trials and pre-post studies), observational studies (e.g., prospective and retrospective cohort studies, case-control studies and analytical cross-sectional studies), and descriptive study designs (e.g., case series, individual case reports, descriptive cross-sectional studies).

#### Exclusion Criteria.

Review articles, commentaries, opinion pieces and conference abstracts will not be included.

## Methods

The scoping review methodology is the most appropriate approach for this study because it seeks to address a broad research question and explore the breadth and depth of the available evidence in an emerging field of research. The proposed scoping review will systematically identify and map relevant studies to provide a comprehensive overview of the current literature. Unlike a systematic review it will not exclude studies based on quality, as the aim is to describe the extent and nature of the evidence rather than assess effectiveness. This protocol was developed in accordance with the guidance and template developed by Lely and Morris et al and is reported in line with the Preferred Reporting Items for Systematic review and Meta-Analysis extension for Protocols (PRISMA-P) guidance [26]. The PRISMA protocol checklist can be found in online supplemental S1 Fig. The scoping review will be conducted using the framework developed by Arksey and O’Malley, with further guidance provided by Levac et al and will align with the recommendations of the Joanna Briggs Institute for elaborating scoping reviews [27–29]. The scoping review will be reported in accordance with the Preferred Reporting Items for Systematic review and Meta-Analysis extension for Scoping Reviews (PRISMA-ScR) [30]. The PCC (Population, concept, context) framework, as recommended by the JBI Manual for Evidence Synthesis, guided the development of the inclusion and exclusion criteria for this protocol.

### Search strategy

A three-step search strategy will be utilised in this review. An initial search of PubMed and CINAHL (EBSCO) using keywords was undertaken to identify relevant articles on the topic. The text words contained in the titles and abstracts of relevant articles, and the index terms used to describe the articles were used to develop a full search strategy. The peer review of electronic search strategy (PRESS) guideline was utilised to guide the creation of the search strategy [31] A senior health science librarian at Atlantic Technological University was consulted to further refine the search strategy. The search strategy is structured around two core concepts: (1) adolescents (aged 10–19 years) and (2) digital health literary or e-health literacy. The search strategy combined controlled vocabulary terms (e.g., MeSH terms such as “Adolescent” and “Health Literacy”) with free-text keywords related to digital health literacy (e.g., “digital health literacy”, e-health literacy”, mHealth literacy”, “health apps”). Boolean operators (AND/OR) were used to combine concepts. Searches were conducted across five electronic databases: PubMed, CINAHL, Web of Science, Scopus and Education Source. No language or date restrictions were applied to the searches. See online supplemental S2 Fig for the full search strategy. In addition, the reference lists of included articles will be manually screened to identify additional relevant records.

### Source of evidence selection

Following the database search, all identified citations were collated and uploaded into Covidence software (Covidence, 2025) and duplicates were removed. Following a pilot test, titles and abstracts are being screened for assessment against the inclusion criteria for the review. Full texts of all potentially relevant citations will subsequently be assessed in detail against the pre-defined inclusion criteria. Reasons for exclusion at the full text review stage will be recorded and reported in the final scoping review. Following the completion of this stage, the reference lists of included studies will be manually searched to identify any articles for inclusion that were not uncovered in the database search.

Due to resource constraints, screening will be conducted primarily by the lead reviewer. To enhance rigour and minimise bias, a blinded independent review of a subset of records will be conducted at both the title and abstract and full-text screening stages by the co-authors. Any discrepancies will be resolved through discussion until a consensus is reached. This approach is consistent with guidance from the JBI and Levac et al., which recognise that scoping reviews allow flexibility in screening procedures while emphasising transparency and strategies to enhance rigour and reduce selection bias.[28,29] The results of the search and the study inclusion process will be reported in full in the final scoping review and presented using a PRISMA flow diagram [30]. The screening stage is expected to be completed by December 2025.

### Data extraction

Data will be extracted using a data extraction form developed by the reviewers, informed by the JBI Manual for Evidence Synthesis [32]. The data extraction form will include the following details: author information (title, author and year of publication), country the study was conducted in, aims and objectives of the study, study design (quantitative, qualitative or mixed methods), type of study (randomised control trial, observational study etc.), measurement tool for digital health literacy (describe), definition of digital health literacy referenced, setting of study (describe), average age of participants, age range of participants, gender of participants, type of population (clinical or non-clinical population) and specific diagnosis or defined subgroup (e.g., neurodevelopmental conditions). The data charting table will be pilot tested on five included studies to ensure consistency between reviewers and shared understanding of the data extraction form. Following discussion and refinement of the extraction categories, data extraction will be conducted by the primary author and independently verified for accuracy and completeness by a second reviewer. Any discrepancies will be resolved through discussion and consensus. The data charting table will be continuously reviewed in an iterative process as recommended by Levac et al. [28]. Any adjustments made to the data extraction form will be detailed and justified in the scoping review. Authors of papers will be contacted where required to request any data or information that is missing in the publication of their study. Data extraction will be performed using Microsoft Excel. Data extraction is expected to be started in December 2025 and finished by the end of January 2026.

### Data analysis and presentation

The literature search, screening and selection process results will be presented narratively and using the PRISMA flowchart. The presentation of results will adhere to the PRISMA-ScR guidelines and will be presented descriptively and in tabular format. Any deviations from this protocol made during the review will be clearly outlined and justified. Results are expected to be generated by the end of February 2026. Descriptive summary statistics will be used to map the characteristics of the included studies. These will include frequency counts and geographic distribution and, where appropriate, by year of publication, study design and sample size. Participant characteristics, including age and gender, will be described narratively and presented graphically where relevant of the participants of the included studies will be described narratively and presented graphically where relevant. A structured overview will also be provided of how digital health literacy is defined in the included studies, the conceptual frameworks underpinning these definitions, the measurement tools and approaches employed. The evolution of definitions and measurement tools over time will be examined where possible.

For the purposes of this review, definition will refer to the explicit author-stated definition of digital health literacy or e-health literacy provided within each study. Conceptualisation will refer to the theoretical frameworks or models underpinning the construct (e.g., multidimensional frameworks or adaptations of existing literacy models). Measurement will refer to the instruments or tools used to operationalise digital health literacy, including the structure of the instrument (e.g., domains assessed, scoring approach) and any reported validation processes. Measurement tools will be descriptively mapped and compared to examine consistency, adaptation, and evolution over time.

## Discussion

This protocol outlines a proposed scoping review that aims to synthesise available evidence on the digital health literacy of adolescents. The review will map how digital health literacy has been defined and measured in the literature with the aim of improving the conceptual clarity within this emerging field. By providing a comprehensive and structured overview of the definitions, underlying conceptual frameworks and measurement tools used with adolescent populations the results will help to identify areas of conceptual fragmentation and inconsistencies in measurement approaches. Mapping the characteristics of included studies will allow identification of gaps in the literature, including underrepresented geographical regions, population subgroups and research contexts. This may inform future empirical research and scale development. It may serve a valuable resource supporting educators and public health practitioners when choosing appropriate measurement tools and designing targeted interventions to support and enhance digital health literacy specifically for this demographic.

Despite these anticipated contributions, several limitations are acknowledged. By only including records published in English, relevant articles published in other languages may be excluded, limiting the comprehensiveness of the review. Due to resource constraints, screening will mainly be conducted by a single reviewer. However, a blinded peer review of a subset of screening decisions will be conducted during the title and abstract screening and the full text screening stages by the two co-authors to minimise selection bias and enhance methodological rigour. A key strength of this review is the comprehensive search strategy that was developed with the assistance of a health science research librarian and informed by the PRESS guideline. The search was conducted across five academic databases and will be supplanted with citation searching to identify any additional records that were not uncovered in the original database search. The inclusion of a broad range of study designs and methods in the proposed scoping review will provide a comprehensive overview of this emerging research area.

The results of the proposed review will be disseminated through publication in a peer-reviewed journal and presentations at relevant academic and public health conferences. In addition, a summary of the findings will be prepared for dissemination through the Atlantic Technological University (ATU) Policy Brief Series to support knowledge translation for policymakers and practitioners. The results will also be shared with stakeholders in education and public health where appropriate, with the aim of informing future research, policy development, and intervention design. As the proposed review forms part of a doctoral research programme, the findings will guide subsequent empirical work in this area.

## Supporting information

S1 Fig PRISMA-P checklist. (DOCX)

S2 Fig Search strategy. (DOCX)

## References

[1] Sørensen K, Van den Broucke S, Fullam J, Doyle G, Pelikan J, Slonska Z, et al. Health literacy and public health: a systematic review and integration of definitions and models. BMC Public Health. 2012;12:80. doi: 10.1186/1471-2458-12-80 22276600 PMC3292515

[2] Nutbeam D. Health literacy as a public health goal: a challenge for contemporary health education and communication strategies into the 21st century. Health Promotion International. 2000;15(3):259–67. doi: 10.1093/heapro/15.3.259

[3] Fleary SA, Joseph P, Pappagianopoulos JE. Adolescent health literacy and health behaviors: A systematic review. J Adolesc. 2018;62:116–27. doi: 10.1016/j.adolescence.2017.11.010 29179126

[4] Coughlin SS, Vernon M, Hatzigeorgiou C, George V. Health Literacy, Social Determinants of Health, and Disease Prevention and Control. J Environ Health Sci. 2020;6(1). 33604453 PMC7889072

[5] Sørensen K, Pelikan JM, Röthlin F, Ganahl K, Slonska Z, Doyle G, et al. Health literacy in Europe: comparative results of the European health literacy survey (HLS-EU). Eur J Public Health. 2015;25(6):1053–8. doi: 10.1093/eurpub/ckv043 25843827 PMC4668324

[6] Lopez Claude KB, Sacks K. Health literacy in the United States Enhancing assessments. United States: Milken Institute. 2022.

[7] van der Vaart R, Drossaert C. Development of the Digital Health Literacy Instrument: Measuring a Broad Spectrum of Health 1.0 and Health 2.0 Skills. J Med Internet Res. 2017;19(1):e27. doi: 10.2196/jmir.6709 28119275 PMC5358017

[8] Pew Research Center. Teens, Social Media and AI Chatbots 2025. 2025.

[9] Boniel-Nissim M, Marino C, Galeotti T, Blinka L, Ozoliņa K, Craig W, et al. A focus on adolescent social media use and gaming in Europe, central Asia and Canada: Health Behaviour in School-aged Children international report from the 2021/2022 survey. Copenhagen: World Health Organization. Regional Office for Europe; 2024.

[10] Stars I, Rubene Z. A Phenomenographic Study of Adolescents’ Conceptions of Health Information Appraisal as a Critical Component of Adolescent Health Literacy. ActPaed. 2020;44:62–80. doi: 10.15388/actpaed.44.5

[11] Park E, Kwon M. Health-Related Internet Use by Children and Adolescents: Systematic Review. J Med Internet Res. 2018;20(4):e120. doi: 10.2196/jmir.7731 29615385 PMC5904452

[12] Hausmann JS, Touloumtzis C, White MT, Colbert JA, Gooding HC. Adolescent and Young Adult Use of Social Media for Health and Its Implications. J Adolesc Health. 2017;60(6):714–9. doi: 10.1016/j.jadohealth.2016.12.025 28259620 PMC5441939

[13] Armstrong M, Halim NK, Raeside R, Jia SS, Hyun K, Boroumand F, et al. How Helpful and What Is the Quality of Digital Sources of Healthy Lifestyle Information Used by Australian Adolescents? A Mixed Methods Study. Int J Environ Res Public Health. 2021;18(23):12844. doi: 10.3390/ijerph182312844 34886569 PMC8657837

[14] Chong YY, Cheng HY, Chan HYL, Chien WT, Wong SYS. COVID-19 pandemic, infodemic and the role of eHealth literacy. Int J Nurs Stud. 2020;108:103644. doi: 10.1016/j.ijnurstu.2020.103644 32447127 PMC7255119

[15] Seidl K, Stauch L, Affengruber L, Sommer I, Wahl A, Rojatz D, et al. Conceptualization of health literacy from the perspective of children and adolescents - a meta-ethnography. Sci Rep. 2025;15(1):5697. doi: 10.1038/s41598-025-89371-9 39962102 PMC11833097

[16] Sawyer SM, Afifi RA, Bearinger LH, Blakemore S-J, Dick B, Ezeh AC, et al. Adolescence: a foundation for future health. Lancet. 2012;379(9826):1630–40. doi: 10.1016/S0140-6736(12)60072-5 22538178

[17] Viner R, Macfarlane A. Health promotion. BMJ. 2005;330(7490):527–9. doi: 10.1136/bmj.330.7490.527 15746135 PMC552817

[18] Casey BJ, Jones RM, Hare TA. The adolescent brain. Ann N Y Acad Sci. 2008;1124:111–26. doi: 10.1196/annals.1440.010 18400927 PMC2475802

[19] Raeside R. Advancing adolescent health promotion in the digital era. Health Promot Int. 2025;40(2):daae172. doi: 10.1093/heapro/daae172 40037909 PMC11879647

[20] Rakić JG, Hamrik Z, Dzielska A, Felder-Puig R, Oja L, Bakalár P, et al. A focus on adolescent physical activity, eating behaviours, weight status and body image in Europe, central Asia and Canada: Health behaviour in school-aged children international report from the 2021/2022 survey. Copenhagen: World Health Organization. 2024.

[21] Költő A, de Looze M, Jåstad A, Nealon Lennox O, Currie D, Gabhainn SN. A focus on adolescent sexual health in Europe, central Asia and Canada: Health behaviour in school-aged children international report from the 2021/2022 survey. Copenhagen: World Health Organization. 2024.

[22] Charrier L, van Dorsselaer S, Canale N, Baska T, Kilibarda B, Comoretto RI, et al. A focus on adolescent substance use in Europe, central Asia and Canada. Copenhagen: World Health Organization. 2024.

[23] Ban S, Kim Y, Seomun G. Digital health literacy: A concept analysis. Digit Health. 2024;10. doi: 10.1177/20552076241287894 39381807 PMC11459536

[24] World Health Organisation. Adolescent health 2025. https://www.who.int/health-topics/adolescent-health?utm_source=chatgpt.com#tab=tab_1. Accessed 2025 August 9.

[25] Norman CD, Skinner HA. eHealth Literacy: Essential Skills for Consumer Health in a Networked World. J Med Internet Res. 2006;8(2):e9. doi: 10.2196/jmir.8.2.e9 16867972 PMC1550701

[26] How to write a scoping review protocol: guidance and template. http://example.com. 2023.

[27] Arksey H, O’Malley L. Scoping studies: towards a methodological framework. International Journal of Social Research Methodology. 2005;8(1):19–32. doi: 10.1080/1364557032000119616

[28] Levac D, Colquhoun H, O’Brien KK. Scoping studies: advancing the methodology. Implement Sci. 2010;5:69. doi: 10.1186/1748-5908-5-69 20854677 PMC2954944

[29] Peters MDJ, Marnie C, Tricco AC, Pollock D, Munn Z, Alexander L, et al. Updated methodological guidance for the conduct of scoping reviews. JBI Evid Synth. 2020;18(10):2119–26. doi: 10.11124/JBIES-20-00167 33038124

[30] Tricco AC, Lillie E, Zarin W, O’Brien KK, Colquhoun H, Levac D, et al. PRISMA Extension for Scoping Reviews (PRISMA-ScR): Checklist and Explanation. Ann Intern Med. 2018;169(7):467–73. doi: 10.7326/M18-0850 30178033

[31] McGowan J, Sampson M, Salzwedel DM, Cogo E, Foerster V, Lefebvre C. PRESS Peer Review of Electronic Search Strategies: 2015 Guideline Statement. Journal of Clinical Epidemiology. 2016;75:40–6. 27005575 10.1016/j.jclinepi.2016.01.021

[32] Aromataris ELC, Porritt K, Pilla B, Jordan Z. JBI manual for evidence synthesis. 2024.

