# Supplementary material for: Digital health literacy of adolescents: A scoping review protocol
Source: PLoS One. 2026 May 6;21(5):e0348021. doi: 10.1371/journal.pone.0348021 (PMC13148673; doi:10.1371/journal.pone.0348021)
Supplement: S2 Fig — (DOCX) [file pone.0348021.s002.docx]

# **Supporting Information**

**S2 Fig. Search Strategy.**

Search strategy completed on 01/05/2025

Web of Science

| Search terms | Results |
| --- | --- |
| TI=("Adolescence" OR "Adolescents" OR "Adolescent" OR "Youth" OR "Youths" OR "Teens" OR "Teenagers" OR "Teenager" OR "pre-adolescen*”) | 410,076 |
| AB=("Adolescence" OR "Adolescents" OR "Adolescent" OR "Youth" OR "Youths" OR "Teens" OR "Teenagers" OR "Teenager" OR "pre-adolescent”) | 467,982 |
| TI=(("Health Literacy" OR "digital health literacy" OR "digital health" OR "digital literacy" OR "ehealth literacy" OR "e-health literacy" OR "electronic health literacy" OR "mhealth literacy" OR "mobile health literacy" OR "mobile health technology” OR "media health literacy" OR "digital health information" OR "online health information" OR "mobile health" OR "health-related information" OR "digital health information" OR “mobile applications” OR “mobile apps” OR "health apps" OR "health application*" OR "health technolog*")) | 27,902 |
| AB=(("Health Literacy" OR "digital health literacy" OR "digital health" OR "digital literacy" OR "ehealth literacy" OR "e-health literacy" OR "electronic health literacy" OR "mhealth literacy" OR "mobile health literacy" OR "mobile health technology” OR "media health literacy" OR "digital health information" OR "online health information" OR "mobile health" OR "health-related information" OR "digital health information" OR “mobile applications” OR “mobile apps” OR "health apps" OR "health application*" OR "health technolog*")) | 55,891 |
| #1 OR #2 | 634,787 |
| #3 OR #4 | 66,456 |
| #5 AND #6 | 3,061 |

CINAHL

| Search terms | Results |
| --- | --- |
| (MH "Adolescence") OR XB ("Adolescence" OR "Adolescents" OR "Adolescent" OR "Youth" OR "Youths" OR "Teens" OR "Teenagers" OR "Teenager" OR "pre-adolescen*") | 704,285 |
| (MH “Mobile applications”) OR (MH “Health literacy”) OR (MH “Digital Health”) OR XB ("Health Literacy" OR "digital health literacy" OR "digital health" OR "digital literacy" OR "ehealth literacy" OR "e-health literacy" OR "electronic health literacy" OR "mhealth literacy" OR "mobile health literacy" OR "mobile health technology" OR "media health literacy" OR "digital health information" OR "online health information" OR "mobile health" OR "health-related information" OR "digital health information" OR “mobile applications” OR “mobile apps” OR “health apps” OR “health application*” OR “health technolog*” | 65,889 |
| S1 AND S2 | 6,521 |

Education source

| Search terms | Results |
| --- | --- |
| TI ("Adolescence" OR "Adolescents" OR "Adolescent" OR "Youth" OR "Youths" OR "Teens" OR "Teenagers" OR "Teenager" OR "pre-adolescen*”) | 124,934 |
| AB ("Adolescence" OR "Adolescents" OR "Adolescent" OR "Youth" OR "Youths" OR "Teens" OR "Teenagers" OR "Teenager" OR "pre-adolescent”) | 171,516 |
| TI (("Health Literacy" OR "digital health literacy" OR "digital health" OR "digital literacy" OR "ehealth literacy" OR "e-health literacy" OR "electronic health literacy" OR "mhealth literacy" OR "mobile health literacy" OR "mobile health technology” OR "media health literacy" OR "digital health information" OR "online health information" OR "mobile health" OR "health-related information" OR "digital health information" OR “mobile applications” OR “mobile apps” OR "health apps" OR "health application*" OR "health technolog*")) | 2,924 |
| AB (("Health Literacy" OR "digital health literacy" OR "digital health" OR "digital literacy" OR "ehealth literacy" OR "e-health literacy" OR "electronic health literacy" OR "mhealth literacy" OR "mobile health literacy" OR "mobile health technology” OR "media health literacy" OR "digital health information" OR "online health information" OR "mobile health" OR "health-related information" OR "digital health information" OR “mobile applications” OR “mobile apps” OR "health apps" OR "health application*" OR "health technolog*")) | 7,695 |
| S1 AND S2 | 218,972 |
| S3 AND S4 | 8,317 |
| S5 AND S6 | 538 |

PubMed

| Search terms | Results |
| --- | --- |
| "Mobile Applications"[MeSH Terms] OR "Health Literacy"[MeSH Terms] OR "Digital Health"[MeSH Terms] OR “digital health literacy"[Title/Abstract] OR "digital health"[Title/Abstract] OR "digital literacy"[Title/Abstract] OR "ehealth literacy"[Title/Abstract] OR "e-health literacy"[Title/Abstract] OR "electronic health literacy"[Title/Abstract] OR "mhealth literacy"[Title/Abstract] OR "mobile health literacy"[Title/Abstract] OR "mobile health technology"[Title/Abstract] OR "Health Literacy"[Title/Abstract] OR "media health literacy"[Title/Abstract] OR "digital health information"[Title/Abstract] OR "online health information"[Title/Abstract] OR "mobile health"[Title/Abstract] OR "health-related information"[Title/Abstract] OR "digital health information"[Title/Abstract] OR “mobile applications”[Title/Abstract] OR “mobile apps”[Title/Abstract] OR “health apps”[Title/Abstract] OR “health application*”[Title/Abstract] OR “health technolog*”[Title/Abstract] | 62,669 |
| "Adolescent"[MeSH Terms] OR "Adolescence"[Title/Abstract] OR "Adolescents"[Title/Abstract] OR "Adolescent"[Title/Abstract] OR "Youth"[Title/Abstract] OR "Youths"[Title/Abstract] OR "Teens"[Title/Abstract] OR "Teenagers"[Title/Abstract] OR "Teenager"[Title/Abstract] OR "pre-adolescen*"[Title/Abstract] | 2,453,664 |
| #1 AND #2 | 7,341 |

Scopus

| Search terms | Results |
| --- | --- |
| TITLE-ABS ("Adolescence" OR "Adolescents" OR "Adolescent" OR "Youth" OR "Youths" OR "Teens" OR "Teenagers" OR "Teenager" OR "preadolescent”) | 757,055 |
| TITLE-ABS ("Health Literacy" OR "digital health literacy" OR "digital health" OR "digital literacy" OR "ehealth literacy" OR "e-health literacy" OR "electronic health literacy" OR "mhealth literacy" OR "mobile health literacy" OR "mobile health technology" OR "media health literacy" OR "digital health information" OR "online health information" OR "mobile health" OR "health-related information" OR "digital health information" "mobile applications" OR "mobile apps" OR "health apps" OR "health application*" OR "health technolog*") | 8,459 |
| S1 AND S2 | 350 |
